# A novel, multiplexed, probe-based quantitative PCR assay for the soybean root- and stem-rot pathogen, *Phytophthora sojae*, utilizes its transposable element

**DOI:** 10.1371/journal.pone.0176567

**Published:** 2017-04-25

**Authors:** James S. Haudenshield, Jeong Y. Song, Glen L. Hartman

**Affiliations:** 1 United States Department of Agriculture-Agricultural Research Service, and Department of Crop Sciences, University of Illinois at Urbana-Champaign, Urbana, Illinois, United States of America; 2 Department of Crop Sciences, University of Illinois at Urbana-Champaign, Urbana, Illinois, United States of America; Agriculture and Agri-Food Canada, CANADA

## Abstract

Phytophthora root rot of soybean [*Glycine max* (L.) Merr.] is caused by the oomycete *Phytophthora sojae* (Kaufm. & Gerd.). *P*. *sojae* has a narrow host range, consisting primarily of soybean, and it is a serious pathogen worldwide. It exists in root and stem tissues as mycelium, wherein it can form oospores which subsequently germinate to release motile, infectious zoospores. Molecular assays detecting DNA of *P*. *sojae* are useful in disease diagnostics, and for determining the presence of the organism in host tissues, soils, and runoff or ponded water from potentially infested fields. Such assays as published have utilized ITS sequences from the nuclear ribosomal RNA genes in conventional PCR or dye-binding quantitative PCR (Q-PCR) but are not amenable to multiplexing, and some of these assays did not utilize control strategies for type I or type II errors. In this study, we describe primers and a bifunctional probe with specificity to a gypsy-like retroelement in the *P*. *sojae* genome to create a fluorogenic 5’-exonuclease linear hydrolysis assay, with a multiplexed internal control reaction detecting an exogenous target to validate negative calls, and with uracil-deglycosylase-mediated protection against carryover contamination. The assay specifically detected 13 different *P*. *sojae* isolates, and excluded 17 other *Phytophthora* species along with 20 non-*Phytophthora* fungal and oomycete species pathogenic on soybean. A diagnostic limit of detection of 34 fg total *P*. *sojae* DNA was observed in serial dilutions, equivalent to 0.3 genome, and a practical detection sensitivity of four zoospores per sample was achieved, despite losses during DNA extraction.

## Introduction

*Phytophthora sojae* (Kaufm. & Gerd.) is an oomycete rotter of soybean [*Glycine max* (L.) Merr.], causing seed decay, root rot, pre- and post-emergence damping off, stem rot, and sometimes foliar blight [1,2]. Although other decay organisms, such as *Pythium* sp., can damage soybean, soybean is the only major host damaged by *P*. *sojae* [3], and losses occur in most soybean-growing regions. Mycelium invades seed, root or other tissues and, although homothallic, can later form sexual *in situ* oospores in tolerant and susceptible cultivars [4] which can survive freezing and thus act as overwintering structures. Eventually the oospores germinate to form sporangia which then release uninucleate zoospores, the major infectious asexual repeating propagules [2]. Zoospores can remain motile for hours, before encysting and germinating to produce hyphae or additional sporangia. The oomycete can also be recovered from infected seeds, which has been considered a route of long-distance disease movement [5]. Disease control methods include the use of genetic resistance in soybean, provided by a series of *Rps* genes [1,6,7], systemic fungicides, fungicidal seed treatments, and improved soil drainage, where possible [1].

Classical methods of identifying *P*. *sojae* through the use of baiting, cultivation on semiselective media, isolating from bacteria, and differentiating from similar oomycetes such as *Pythium* sp. [8], can be cumbersome, time-consuming, and esoteric, making meaningful quantification difficult. Thus, the availability of methods of molecular analysis of the genetic sequences are attractive as potential diagnostic tools to determine the presence and quantity of target sequences in crop residue, soils, and water sources.

Previous molecular work has often focused on the ITS sequences from the nuclear ribosomal RNA genes. One conventional PCR assay [9] developed for *P*. *sojae* utilized primers PS1 and PS2, to produce a 330 bp amplicon, but specificity was reported to be somewhat sensitive to shifts in the annealing temperature, potentially leading to type I errors. Nevertheless, the authors used these primers to develop a SYBR-green based Q-PCR protocol with an apparent 10 pg diagnostic limit of detection. Subsequently another SYBR-green based assay was developed [10], also targeting the ITS region of the *P*. *sojae* genome, using primers PSOJF1 and PSOJR1 to produce a 127 bp product. They found specificity and sensitivity to a diagnostic limit of detection of 1 pg DNA. Other researchers [11] using these primers reported diagnostic limit of detection of 10 fg in absolute quantifications. In this instance, the authors reported on performing parallel SYBR-green assays for a host sequence, and having used the comparative ∆Ct method to normalize to host DNA and to check for inhibitors (which could lead to type II errors) in the template preparations. Other genetic targets described for *P*. *sojae* detection include a Ras-related protein coding gene for loop-mediated isothermal amplification [12] and a *A3aPro* transposon-like element for standard PCR and nested PCR [13]. These illustrate the usefulness of alternative, non-rDNA sequences, for qualitative diagnostics and potentially quantitative analyses.

Dye-binding (SYBR green-type) Q-PCR assays are convenient because they may sometimes be adapted from existing conventional PCR assays, yet provide the possibility for quantitation. However, they are not amenable to multiplexing, because any amplification product will produce a fluorescent signal indistinguishable from that of other products. Probe-based assays, such as the fluorogenic 5’-exonuclease linear hydrolysis assay (Taqman®) offers the potential for increased specificity, SNP-differentiation, and multiplexing with compatible assays using a variety of fluorescent reporters. Drawbacks include modestly increased cost, the general need for an amplified sequence of under *ca*. 300 bp, and the need for a conserved sequence, of appropriate melting point, between the two primers where a fluorogenic probe can anneal to all targeted isolates of the desired taxon. The challenge, as always, is to identify the target sequence meeting both the diagnostic criteria and the biochemical demands [14]. For example, some genetic targets for which primers are available, such as the avirulence gene *Avr1c* [15], could be useful in situations where one is only interested in detecting the specific isolates carrying that trait, rather than members of the entire species.

Because the rRNA sequences of the *P*. *sojae* genome have proven challenging for the purposes of developing a selective and sensitive Q-PCR assay, we turned to another target sequence resembling the *Ty3*/*Gypsy* retroelement that has been reported to be widely distributed in the *Phytophthora* genus and to form lineages that predate the speciation events of the genus [16]. This retroelement could make it an ideal candidate for specifically identifying *P*. *sojae*, because it would be present in every isolate, and in multiple copies per genome. Conventional PCR primers PS12 and PS6R were developed to target this element and were validated to produce a 282 bp amplicon in all 24 isolates of *P*. *sojae* tested, and to exclude 17 non-*sojae* species of *Phytophthora*, as well as 16 other fungal pathogens of soybean, and soybean itself [17].

We report here the development of a novel, probe-based Q-PCR assay for *P*. *sojae*, and a multiplexed internal control reaction to validate negative calls, targeting the gypsy-like retroelement present in the *P*. *sojae* genome. Partial results using the conventional PCR primers and the Q-PCR assay were previously published in abstracts [17,18].

## Materials and methods

DNA was purified from mycelium of *P*. *sojae* isolate 1–23 grown in dilute V8 juice liquid cultures or scraped from dilute V8 juice solid medium, and from zoospores on water-agar, using the FastDNA Spin Kit of MP Biomedicals (Solon-OH), per the manufacturer’s instruction, and using Lysing Matrix A and the CLS-Y extraction buffer option. Tissue disruption was performed at room temperature in a lemniscate bead beater (FastPrep-120; MP Biomedicals) at a speed of 6 m/s for 40 s with a one-hour incubation on ice after homogenization followed by a second round of homogenization. The DNAs and amplicons for sequencing were further purified using the QiaQuick clean-up kit (Qiagen, Valencia-CA) and quantified spectrophotometrically. Ten-fold serial dilutions of *P*. *sojae* (isolate 1–23) DNA spanning 7 log orders of magnitude (0.67 ng/μl to 0.67 fg/μl) were prepared in 5 mM Tris, pH 8, containing 1 μg/ml sheared salmon DNA (Ambion, Austin-TX) as a carrier, in siliconized microcentrifuge tubes. Aliquots of each concentration were frozen at -80°C and thawed as needed for use as absolute quantification standards. Given an estimated 95 Mb haploid genome size for *P*. *sojae* [19], the dilutions series therefore ranged from *ca*. 6,500 genomes per μl to 0.0065 genomes per μl.

Primers Pso12-F and Pso6-R (Table 1) were synthesized by Integrated DNA Technologies (IDT, Coralville-IA) with standard desalting purification. The primers were used to produce a 282 bp amplicon from DNA of isolate 1–23 by conventional PCR using Phusion high-fidelity DNA polymerase (New England Biolabs, Ipswich-MA). The amplicon was bidirectionally sequenced three times at two different core facilities, using the same primers and Sanger chemistry. Nucleotide sequence data were aligned and a consensus sequence was determined manually and deposited to GenBank (accession number KY315993). Fragment analysis of the amplicon (made with a 5’-HEX-labeled Pso12-F primer) was conducted on a model AB3730xl capillary DNA analyzer (Thermo-Fisher, Pittsburgh-PA) with a ROX 400 standard, at the Roy J. Carver Biotechnology Center Core Sanger Facility (University of Illinois, Urbana-Champaign). From the consensus sequence, a probe (Pso-P5, Table 1) was selected using the online PrimerQuest tool provided by IDT (www.idtdna.com), and synthesized by the same as a dual-labeled probe with the FAM reporter at the 5’ end and the IowaBlack quencher at the 3’ end, with HPLC purification. An internal control reaction was used to validate any negative diagnostic calls, with a dual-labeled probe (CoreIC-Cy5) and exogenous target oligo (PsoIC-seq) (Table 1) designed in the manner previously described [20]. The exogenous target was diluted to prepare a 1 fM stock solution.

**Table 1:** Oligonucleotide sequences used for Q-PCR.

Oligo	Sequence
Pso12-F	5’-CAGGT TTTCA GCGAT CTCAT CCAAG TG-3’
Pso6-R	5’-CACAT TGCGG AAAAG GAGGT GATTG CT-3’
Pso-P5	5’-fam-TGCCG ACTGC GAGGT CAGCA ACCAC TTCAA-ibfq-3’
CoreIC-Cy5	5’-cy5-TGCTT AGGAC GAGAA CTCCC ACATC-ibrq-3’
PsoIC-seq	5’-CAGGT TTTCA GCGAT CTCAT CCAAG TGCAT GCTTA GGACG AGAAC TCCCA CATCG AGCTG GACAT CTGCA GCAAT CACCT CCTTT TCCGC AATGT G-3’

Fluorescent reporters FAM and CY5 (and the quenchers IBFQ and IBRQ, respectively) are shown on the two probes, but alternatives work as well.

Q-PCR was conducted in a model Mx3005p real-time thermocycler (Agilent, Santa Clara-CA), using Invitrogen Platinum qPCR SuperMix-UDG (Thermo-Fisher, Pittsburgh-PA), with the supplied ROX dye as a passive reference, and including bovine serum albumen (BSA) to reduce potential inhibition [21]. Each reaction contained 12.5 μl SuperMix, 6 mM magnesium chloride (3 mM derived from the SuperMix), 50 nM ROX, 250 nM Pso12-F, 600 nM Pso6-R, 200 nM Pso-P5, 150 nM CoreIC-Cy5, 50 ymol (30 copies) PsoIC-seq, 400 ng/μl BSA, template DNA, and sufficient water to bring the final volume to 25 μl. For each quantification run, to ensure identical reagent composition, non-variable reaction components were precombined in a “cocktail” such that reactions were assembled by addition of a 20 μl cocktail to 5 μl template DNA.

All disposable plasticware, buffers, and water was sterile and certified nuclease-free; pipet tips were aerosol-resistant. The SuperMix incorporated both “hot start” reaction assembly (inactivated polymerase) to prevent primer degradation, and uracil DNA deglycosylase to prevent potential carryover contamination by previously amplified targets. Q-PCR amplification utilized a thermal profile consisting of 2 min at 50°C, followed by 2 min at 95°C, followed by 40 cycles of 95°C for 15 s and 60°C for 30 s. The instrument fluorometer gain settings were adjusted so that baselines and C_T_ values were in within the linear dynamic range of sensitivity. FAM fluorescence, normalized to ROX fluorescence (dRn), was selected for tracking amplifications. The driver software (MxPro v.4.10) was set to use adaptive baselines and to determine the amplification threshhold automatically.

DNAs of 13 *P*. *sojae* isolates, 17 other *Phytophthora* species (generous gift of P. W. Tooley, USDA-ARS, Ft. Detrick, MD, USA), and 20 non-*Phytophthora* fungal and oomycete pathogens of soybean were obtained and quantified spectrophotometrically. The *Phytophthora* spp. DNAs were diluted to 2 ng/μl and the non-*Phytophthora* spp. were diluted to 0.1 ng/μl. As an initial test of specificity, primers Pso12F and Pso6R were used in conventional PCR to amplify the DNAs of the non-*sojae Phytophthora* species along with *P*. *sojae* isolate 1–23, and amplification products were electrophoretically separated in agarose, stained with ethidium bromide, and compared to a DNA reference ladder. After a hydrolysis probe had been designed, duplicate 5 μl samples of all test panel DNAs were subjected to multiplex Q-PCR with the internal control reaction. Similarly, a sample of soybean DNA was subjected to multiplex Q-PCR.

*P*. *sojae* isolate 1–23 growing on water agar and observed to have formed numerous oospores was induced to produce sporangia by the Chen-Zentmeyer salts solution method [8,22] and resulting zoospores were collected and filtered free of mycelial fragments. The zoospores were spread on 1% water agar and observed under a stereoscopic dissecting microscope. Individual zoospores (or cysts) were isolated by excising the supporting agar with a scalpel and transferring the agar and spore to a Lysing Matrix A tube (FastDNA kit). Single spores and multiple spores were thus collected, and then DNA was extracted. A volumetric loss of 60% occurred, and DNA was eluted in a final volume of 20 μl, from which 5 μl subsamples were assayed by Q-PCR.

To evaluate the practical utility of the assay in a screening for disease resistance, germinated seedlings in greenhouse flats were inoculated with mycelial emulsion from agar-grown cultures of races 1, 4, 17, and 28, using the hypocotyl-splitting method [23]. Host genotypes were Williams-OH (*rps*), Williams 82 (*Rps*1k), and Williams (*rps*)—the latter being grown as a non-inoculated susceptible check in each flat. There were 9–11 plants in each treatment. At 3 d post inoculation (DPI), a 5 mm long hypocotyl segment was excised at 1 cm above the point of inoculation, placed in a Lysing Matrix A tube, and total DNA was extracted using the method previously described. Subsamples of the eluates from the FastDNA kit were diluted by a factor of 50 in 5 mM Tris pH 8, and 5 μl samples were assayed in duplicate by Q-PCR for *P*. *sojae* DNA.

## Results

Preliminary amplification by conventional PCR of *P*. *sojae* isolate 1–23 and the other *Phytophthora* species using the Pso12-F and Pso6-R primers showed no discernable products when the reactions were electrophoresed in agarose, except for a single band in the case of *P*. *sojae*. Fragment analysis of the amplicon produced by the *P*. *sojae* template DNA revealed a single size product, which was in agreement with the results of a SYBR-green melting curve analysis (data not shown). Sanger sequencing of the amplicon produced a consensus sequence of 282 nt (GenBank accession number KY315993), from which the 30 nucleotide linear hydrolysis probe Pso5-P was designed.

**Fig 1:**
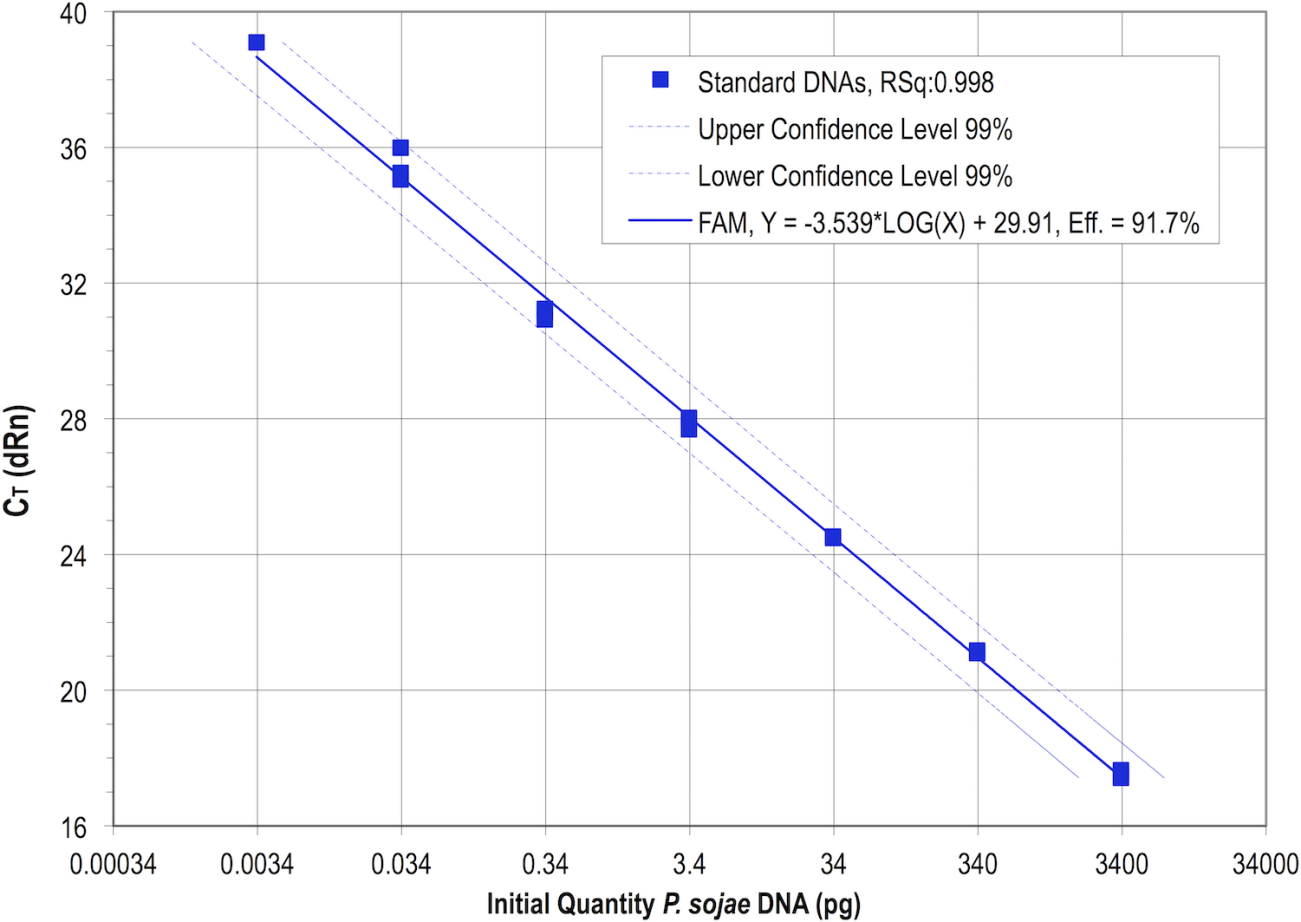
Standard curve and reaction efficiency for *Phytophthora sojae* DNA quantification by Q-PCR. (Samples in triplicate.)

Q-PCR was performed on triplicate 5 μl samples of *P*. *sojae* DNA from the dilution series. When the C_T_ values were plotted on a semilog chart as a function of initial DNA amount (Fig 1), a linear response was observed (coefficient of linear correlation = 0.998.) Reaction efficiency was calculated from the standard curve by the instrument software to be 91.7%. No-template controls containing diluent did not amplify. Two of the three replicates at the 3.4 fg quantity did not amplify, but one did and was plotted as a singleton and included in the analysis. In all other cases, all three reactions gave highly similar results. Because the lowest amount of *P*. *sojae* DNA that always amplified was 34 fg [both in Fig 1 and in other instances where the full dilution series was used (data not shown)], this was set as the diagnostic limit of detection, and is calculated to be about 0.3 genome. When individual or clusters of zoospores were excised from water-agar and DNA was extracted with elution in a final volume of 20 μl, detection of *P*. *sojae* was not achieved from individual spores, but groups of four spores were detectable by Q-PCR (data not shown).

**Fig 2:**
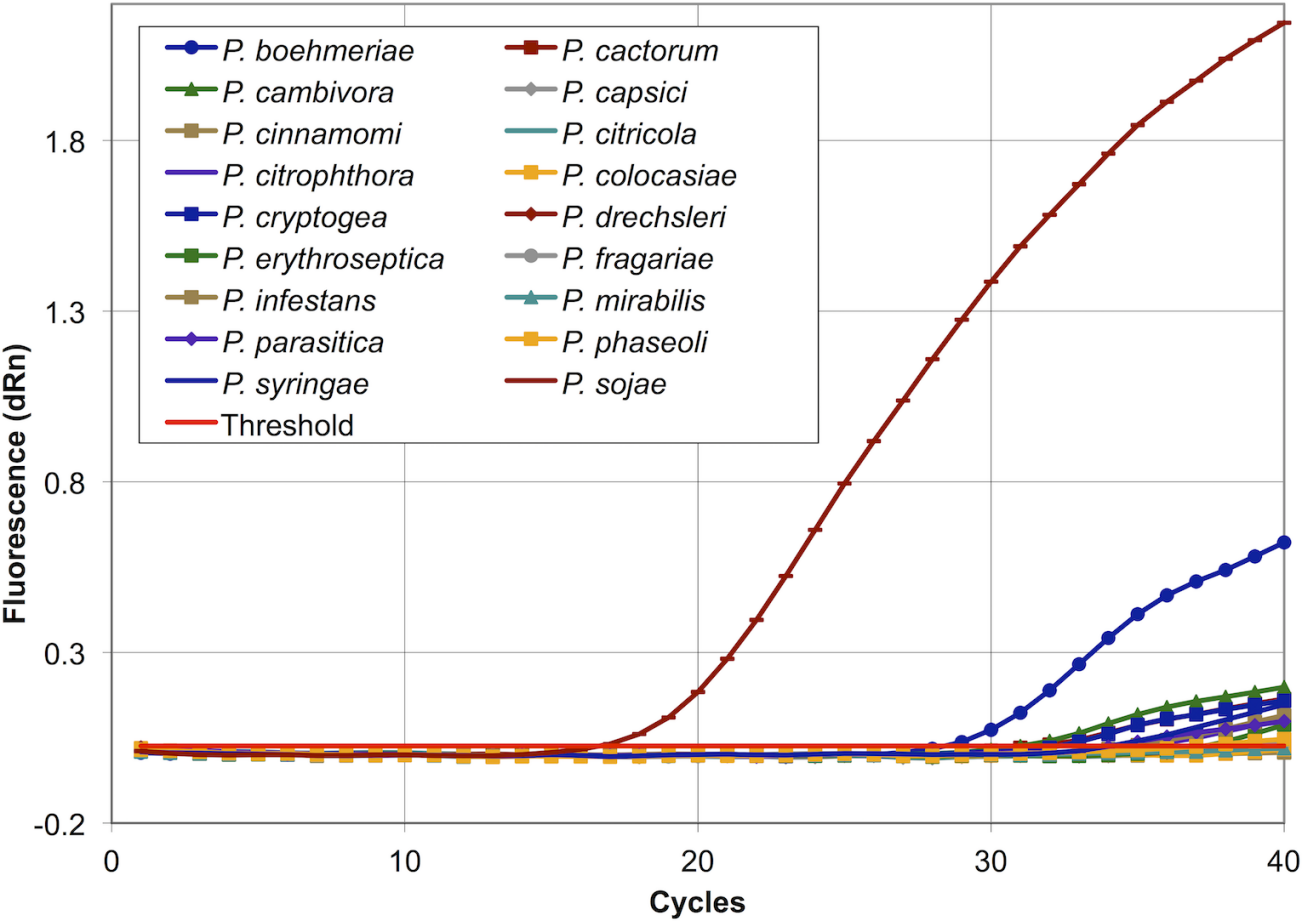
Amplification profiles of *Phytophthora* species.

Laboratory validation DNAs of 13 P. *sojae* isolates, 17 other *Phytophthora* species, 13 non-*Phytophthora* fungal and oomycete pathogens of soybean and of soybean cultivar Williams 82 were evaluated with the new Q-PCR assay. The assay efficiently detected the 13 *P*. *sojae* isolates, with strong amplification roughly equivalent to isolate 1–23, as shown in Table 2. No appreciable amplification of non-*sojae Phytophthora* species was observed except in the case of *P*. *boehmeriae*, which gave a weak signal, less than 0.05% of the observed amplification of *P*. *sojae* itself (Table 3, Fig 2). Only three *Phytophthora* species were slightly amplified to indicate more than the diagnostic limit of detection (*P*. *cactorum*, *P*. *cambivora*, and *P*. *cryptogea*), but each of these was below 0.001% of *P*. *sojae*. In every case the internal control reaction gave the expected positive signal, indicating a functional assay and absence of inhibitors. No amplification was observed for DNAs of 20 non-*Phytophthora* fungal and oomycete pathogens (Table 4). Again, in every case the internal control reaction gave the expected positive signal. To ensure that host DNA would not be detected by the assay, 10 ng of soybean DNA was subjected to Q-PCR, and as expected, no amplification was observed.

**Table 2:** Assay validation with 13 isolates of *Phytophthora sojae*.

Isolate	Race	Ct (dRn)	Quantity (pg)*	Relative %^†^
PS79	28	12.99	28,000	160
PS42	4	13.29	23,000	130
PS62	4	13.33	22,000	110
PS28	28	12.88	30,000	110
PS59	28	13.58	19,000	100
PS56	43	14.17	13,000	81
PS46	17	14.98	7,500	75
PS39	3	14.81	8,400	65
PS73	3	14.57	9,800	61
PS72	3	14.48	10,000	56
PS12	7	13.4	21,000	42
PS71	1	13.26	23,000	30
PS78	26	19.44	390	24

* Quantity means the amount determined by the assay.

^†^ Relative % indicates the quantified amount as a percentage of the template amount. (Negative controls not shown.)

**Table 3:** Assay validation with 17 non-*sojae* species of *Phytophthora* (plus positive control).

Species	Ct (dRn)	Quantity (pg)*
*P*. *boehmeriae*	28.51	1.1
*P*. *cactorum*	31.61	0.13
*P*. *cambivora*	31.04	0.19
*P*. *capsici*	38.19	0.00
*P*. *cinnamomi*	No Ct	0.00
*P*. *citricola*	No Ct	0.00
*P*. *citrophthora*	35.25	0.01
*P*. *colocasiae*	No Ct	0.00
*P cryptogea*	32.38	0.08
*P*. *drechsleri*	38.38	0.00
*P*. *erythroseptica*	37.36	0.00
*P*. *fragariae*	37.42	0.00
*P*. *infestans*	34.82	0.02
*P*. *mirabilis*	No Ct	0.00
*P*. *parasitica*	34.21	0.02
*P*. *phaseoli*	37.19	0.00
*P*. *syringae*	34.1	0.02
*P*. *sojae*	16.71	2,945

* Quantity means the amount determined by the assay.

**Table 4:** Assay validation with 20 non-*Phytophthora* fungal and oomycete pathogens.

Species	Ct (dRn)	Quantity (pg)
*Cadophora gregata*	No Ct	0.00
*Cercospora sojina*	No Ct	0.00
*Colletotrichum truncatum*	No Ct	0.00
*Colletotrichum gloeosporioides*	No Ct	0.00
*Cadophora gregata*	No Ct	0.00
*Diaporthe phaseolorum var*. *sojae*	No Ct	0.00
*Diaporthe phaseolorum var*. *caulivora*	No Ct	0.00
*Diaporthe phaseolorum var*. *meridionalis*	No Ct	0.00
*Erysiphe polygoni*	No Ct	0.00
*Fusarium oxysporum f*. *sp*. *lycopersici*	No Ct	0.00
*Fusarium oxysporum f*. *sp*. *radici-lycopersici*	No Ct	0.00
*Fusarium virguliforme*	No Ct	0.00
*Macrophomina phaseolina*	No Ct	0.00
*Penicillium sp*.	No Ct	0.00
*Phomopsis longicolla*	No Ct	0.00
*Pythium sp*.	No Ct	0.00
*Rhizoctonia solani*	No Ct	0.00
*Sclerotinia sclerotiorum*	No Ct	0.00
*Phakopsora pachyrhizi*	No Ct	0.00
*Stemphylium vesicarium*	No Ct	0.00

In practical assays for resistance in genotypes known to be susceptible or resistant to *P sojae* races 1, 4, 17, and 28, large amounts of pathogen DNA were found in susceptible combinations, at 3 DPI (Table 5). In every non-inoculated check, there was less than 0.01 pg *P*. *sojae* DNA found (only one plant had a trace.). The inoculated *rps* suscept was found to to be well-infected by all four races, with 190–430 pg *P*. *sojae* DNA determined in each assay. The inoculated *Rps*1k genotype was found to be very susceptible to *P*. *sojae* races 4 and 28 (95–330 pg DNA), and highly resistant to races 1 and 17 (0–0.03 pg DNA). Although the standard deviations were relatively high, the differences between resistance and susceptible was large that the assay could readily differentiate the genotypes.

**Table 5:** Amount of *Phytophthora sojae* DNA found in greenhouse-grown, inoculated hypocotyls of soybean.

*P*. *sojae* Race	Host Genotype	n	Quantity (pg)	% SD
1	*rps*	9	360	45
1	*Rps*1k	9	0	0
1 ni	*rps*	10	0	0
4	*rps*	11	230	26
4	*Rps*1k	11	95	140
4 ni	*rps*	9	0	0
17	*rps*	9	190	36
17	*Rps*1k	8	0.03	160
17 ni	*rps*	9	0	0
28	*rps*	10	430	26
28	*Rps*1k	11	330	53
28 ni	*rps*	10	0.01	300

n, Number of replicates. pg DNA, Average picograms of *P*. *sojae* DNA found. NI, Non-inoculated check. % SD, Standard deviation of the average amount of DNA found, expressed as a percentage of that average.

## Discussion

Q-PCR has arisen as a valuable tool in both diagnostic and research laboratories, because of the relative speed of the process and reduced skill set required of analysts, versus the array of conventional methods. The high degree of selectivity and sensitivity offered by many Q-PCR protocols is dependent upon careful validation of the assay, with regard to the sample handling, processing, extraction and purification, and a recognition that the particular sequence being amplified may or may not reflect the situational needs. One worker may wish to include an entire genus in the target class, whereas another worker may need species- or SNP-level resolution. In some cases, a toxin or other gene product may be the defining criterion of the target pathogen. As a result, it is common for multiple Q-PCR assays to be developed for a given species, each having strengths for a particular purpose [24]. In our case, the aim was to develop a quantitative PCR assay amenable to multiplexing, and targeting a genetic sequence capable of providing the species-level discrimination of *P*. *sojae*.

Because the Q-PCR assays currently available for *P*. *sojae* were of the dye-binding type, multiplexing with an exogenous control was not available, which for our purposes was an important consideration because of the potential for inhibitors from soil and plant materials to interfere with detection of the pathogen. Furthermore, there have been difficulties reported [11] with the efficacy of some earlier PCR assays for *Phytophthora*. Additionally, the 127 nt amplicon produced by primers PSOJF1 and PSOJR1 did not contain adequate conserved sequence to permit development of a hydrolysis probe. In choosing the the *Ty3*/*Gypsy* retroelement as a target for developing a fluorogenic 5’ exonuclease linear hydrolysis assay, we hoped to utilize an ancestral sequence common to all *P*. *sojae*, but divergent from other *Phytophthora* sp., and which by virtue of the multi-copy nature of the element, might offer superior sensitivity versus a single-copy gene. We also developed a multiplexed internal control reaction detecting an exogenous target to validate negative calls. Finally, our choice of commercial enzyme mix includes uracil-deglycosylase-mediated protection against carryover contamination, reducing the potential for false positives.

The resulting assay produced standard curves for absolute quantification of *P*. *sojae* DNA with a reference dilution series that routinely showed over 90% amplification efficiency and a coefficient of linear correlation better than 0.995 (Fig 1). Whenever Q-PCR was performed to quantify *P*. *sojae*, freshly-thawed aliquots (in duplicate) of the same dilution series were included on each assay plate so that absolute quantification was effected. Whenever a negative call was made (including for the negative control wells), it was not considered valid unless the multiplex internal control gave the expected amplification signal, typically around cycle 35 (data not shown).

We found the assay to be very sensitive, with a limit of detection of 34 fg total *P*. *sojae* DNA, which was estimated to represent approx. 0.3 haploid genome, assuming a 95 Mb genome size [19]. (An earlier estimate of the *P*. *sojae* genome size reported it to be 62 Mb [25] but this would not substantively change our findings.) Our negative control testwells, whether buffer or diluent, were always completely negative (data not shown). We note that the bead-beating DNA extraction protocol employed is known to fragment genomic DNA, which works in the favor of this type of assay, as it makes the multiple copies of target sequence more uniformly distributed in solution and likely to be present in subsamples of DNA extracted from only a few cells. One drawback, however, is that the number of copies of retroelement may vary somewhat from one species to another [16] and could lead to reduced accuracy in quantifications among, and even within, species; this may have contributed to the range of response we observed among *P*. *sojae* isolates (Table 2).

Individual zoospores were not detected, likely because of a combination of the recovery efficiency during DNA extraction (50%) and the fact that only a portion of the final eluate was assayed in each replicate tube (5 μl of 20). However, four zoospores (or cysts) was adequate to produce a positive result. In resistance screening experiments, the assay proved to be more than adequate for determining infection of plants, as the DNA eluates were diluted 1/50, and yet substantial amounts of fungal DNA representing many millions of genomes could be recovered from some hypocotyl segments when the plant did not carry resistance to the corresponding *P*. *sojae* race.

Our results also showed the assay to be highly exclusive, in that 16 of 17 non-*sojae* species of *Phytophthora* not appreciably detected (Table 3, Fig 2), and 20 non-*Phytophthora* fungal pathogens were not detected at all (Table 4). By using 20 times as much template DNA (10 ng) for *Phytophthora* species, as for non-Phytophthora species (0.5 ng), we intended to “push” the assay, to check for off-target detection. Only one species, *P*. *boehmeriae*, was notably amplified, but comparatively the assay was over two thousand times more sensitive to *P*. *sojae*. Given the specific host, *Ailanthus altissima*, false-positive reactions are unlikely in most situations, but the potential must be borne in mind. Adjusting the cycle threshold or elevating the primer annealing temperatures could potentially mitigate the amplification of it and other poorly-amplified non-targets. Our *Phytophthora* test panel was not exhaustive, however, and there could yet be untested species in soybean fields that might cross react, such as *P*. *sanomeana*.

We have used the assay for production analysis of numerous seed and root system evaluations and found continuing utility in discovery research projects. The assay may be of interest to diagnosticians, soybean breeders, and researchers alike.

## References

[1] Dorrance AE. Phytophthora root and stem rot In: Hartman GL, Rupe JC, Sikora EF, Domier LL, David JA, and Steffy KL, editors. Compendium of Soybean Diseases and Pests, American Phytopathological Society; 2015 pp 73–76.

[2] Hildebrand AA. A root and stalk rot of soybeans caused by *Phytophthora megasperma* Drechsler var. *sojae* var. nov. Can. J. Bot. 1959;37: 927–957.

[3] Jones JP, Johnson HW. Lupine, a new host for *Phytophthora megasperma* var. *sojae*. Phytopathology. 1969; 59: 504–507.

[4] Slusher RL. Sinclair JB. Development of *Phytophthora megasperma* var. *sojae* in soybean roots. Phytopathology. 1973;63: 1168–1171.

[5] Klein HH. Etiology of the *Phytophthora* disease of soybeans. Phytopathology 1959;49: 380–383.

[6] Morgan FL, Hartwig EE. Physiologic specialization in *Phytophthora megasperma* var *sojae*. Phytopathology. 1965;55: 1277–1279.

[7] Athow KL. Fungal diseases. In: Wilcox JR, Boerma HR, Kamprath EJ, Schrader LE, editors. Soybeans: Improvement, Production and Uses. Agron. Monogr. No. 16. ASA-CSSA-SSSA, Madison, WI; 1987. pp 687–727.

[8] Sinclair JB, Dhingra OD. Basic Plant Pathology Methods, 2nd ed. CRC Press, Boca Raton, FL 1985, 355 p.

[9] Wang Y, Zhang W, Wang Y, Zheng X. Rapid and sensitive detection of *Phytophthora sojae* in soil and infected soybeans by species-specific polymerase chain reaction assays. Phytopathology 2006;96: 1315–1321. doi: 10.1094/PHYTO-96-1315 18943663 10.1094/PHYTO-96-1315

[10] Bienapfl JC, Malvick DK, Percich JA. Specific molecular detection of *Phytophthora sojae* using conventional and real-time PCR. Fung. Biol. 2011;115: 733–740. 10.1016/j.funbio.2011.05.007 21802053

[11] Catal M, Erler F, Fulbright DW, Adams G. Real-time quantitative PCR assays for evaluation of soybean varieties for resistance to the stem and root rot pathogen *Phytophthora sojae*. Eur. J. Plant Pathol. 2013;137: 859–869.

[12] Zhao W, Wang T, Qi R. *Ypti* gene-based detection of *Phytophthora sojae* in a loop-mediated isothermal amplification assay. J. Plant Dis. Prot. 2015;122: 66–73.

[13] Dai T-T, Meng J, Dong S, Lu C-C, Ye W, Zheng X, Wang Y-C. A *Phytophthora* conserved transposon-like DNA element as a potential target for soyabean root rot disease diagnosis. Plant Path. 2013; 62: 719–726.

[14] Haudenshield JS, Hartman GL. Molecular methods for pathogen detection and quantification In: Hartman GL, Rupe JC, Sikora EF, Domier LL, David JA, and Steffy KL, editors. Compendium of Soybean Diseases and Pests, American Phytopathological Society; 2015 pp 131–135.

[15] Wen J, Chen Q, Sun L, et al A SCAR marker specific for rapid detection of the avirulence gene *Avr1c* in *Phytophthora sojae*. J. Gen. Plant Pathol. 2014;80: 415–422.

[16] Judelson HS. Sequence Variation and Genomic Amplification of a Family of *Gypsy*-like Elements in the Oomycete Genus *Phytophthora*. Mol. Biol. Evol. 2002;19: 1313–1322. 12140243 10.1093/oxfordjournals.molbev.a004192

[17] Song J Jeon N, Li S, Kim H, Hartman GL. Development of PCR assay using species-specific primers for *Phytophthora sojae* based on the DNA sequence of its transposable element. Phytopathology. 2007;97: S110.

[18] Haudenshield JS, Hartman GL. A multiplexed, probe-based quantitative PCR assay for DNA of *Phytophthora sojae*. Phytopathology. 2010;100: S48. 10.1371/journal.pone.0176567 PMC5404879 28441441

[19] Tyler BM, Tripathy S, Zhang X, Dehal P, Jiang RHY, Aerts A, et al *Phytophthora* genome sequences uncover evolutionary origins and mechanisms of pathogenesis. Science. 2006;313: 1261–1266. doi: 10.1126/science.1128796 16946064 10.1126/science.1128796

[20] Haudenshield JS, Hartman GL. Exogenous controls increase negative call veracity in multiplexed quantitative PCR assays for *Phakopsora pachyrhizi*. Plant Dis. 2011;95: 343–352. 10.1094/PDIS-01-10-0023 30743502

[21] Jiang J., Alderisio KA, Singh A, Xiao L. Development of procedures for direct extraction of *Cryptosporidium* DNA from water concentrates and for relief of PCR inhibitors. Appl. Env. Microb. 2005;71: 1135–1141. 10.1128/AEM.71.3.1135-1141.2005 PMC1065175 15746310

[22] Chen D, Zentmyer GA. Production of Sporangia by *Phytophthora cinnamomi* in Axenic Culture. Mycologia 1970;62: 397–402.

[23] Slaminko TL, Bowen CR, Hartman GL. Multi-year evaluation of commercial soybean cultivars for resistance to *Phytophthora sojae*. Plant Dis. 2010;94: 368–371. 10.1094/PDIS-94-3-0368 30754241

[24] Kandel YR, Haudenshield JS Srour AY, Islam KT, Fakhoury AM., Santos P, et al Multilaboratory comparison of quantitative PCR assays for detection and quantification of *Fusarium virguliforme* from soybean roots and soil. Phytopathology. 2015;105: 1601–1611. doi: 10.1094/PHYTO-04-15-0096-R 26368513 10.1094/PHYTO-04-15-0096-R

[25] Moa Y, Tyler BM. Genome organization of *Phytophthora magasperma* f.sp. *glycinea*. Exp. Mycology. 1991;15: 283–291.

